# Topographic correlation of microperimetry with foveal microstructure characteristics in idiopathic epiretinal membrane patients with an ectopic inner foveal layer

**DOI:** 10.1186/s40662-024-00419-9

**Published:** 2025-02-01

**Authors:** Qianqian Wang, Congyao Wang, Yihua Su, Fenfen Yu, Tingting Chen, Xia Dong, Pengxia Wan

**Affiliations:** Department of Ophthalmology, The First Affiliated Hospital, Sun Yat-Sen University, No. 58 Zhongshan Er Road, Guangzhou, 510080 Guangdong People’s Republic of China

**Keywords:** Microperimetry, Epiretinal membrane, Ectopic inner foveal layer, Vessel density

## Abstract

**Purpose:**

To identify foveal structure-function topographic association and relationship in patients with idiopathic epiretinal membrane (ERM) related to ectopic inner foveal layer (EIFL).

**Methods:**

This was a cross-sectional, observational study that involved 40 individuals with idiopathic ERM: 22 without EIFL (Group 1) and 18 with EIFL (Group 2). Quantitative foveal light sensitivity was measured using microperimetry, and foveal microstructure was assessed using spectral-domain optical coherence tomography (SD-OCT) and optical coherence tomography angiography (OCTA). Multiple indices of microvascular parameters of OCTA images were further processed using the AngioTool software. LASSO regression and quantile regression analyses were performed to identify the spatial distribution correlation between foveal light sensitivity and foveal microstructure parameters.

**Results:**

Group 2 exhibited reduced light sensitivity across all parameters of microperimetry compared to Group 1 (*P* < 0.001). Additionally, the central foveal thickness, the percentage of ellipsoid zone disruption, and the foveal avascular zone area were significantly lower in Group 1 than in Group 2 (all *P* < 0.005). Compared to Group 1, the vessel density (VD) and perfusion density of the foveal region was significantly increased in Group 2 (*P* < 0.001). In contrast, Group 2 showed significantly decreased VD in the parafoveal region compared with Group 1 (*P* < 0.05). Significant differences in OCTA parameters including ‘total number of junctions’, ‘junction density’, ‘total vessel length’, ‘average vessel length’, ‘total number of end points’ were observed between Group 1 and Group 2 (all *P* < 0.01). The foveal light sensitivity was significantly positively correlated with VD in the parafoveal region and negatively correlated with EIFL alteration, best-corrected visual acuity and ellipsoid zone disruption [Log(λ) = − 0.18303, λ = 0.6561].

**Conclusions:**

The presence of EIFL and decreased VD in the parafoveal region, factors that collectively elevate the risk of disease progression, are significantly and independently correlated with reduced microperimetric retinal sensitivity in patients with idiopathic ERM.

## Background

Idiopathic epiretinal membrane (ERM) is characterized by fibrotic proliferation that develops on the inner surface of the neurosensory retina [1]. ERMs exert both centrifugal and contractile forces on the retinal surface, leading to thickening and deformation of the retinal layer structure and subsequently altering the morphology of the inner retinal layers. According to Govetto et al.’s staging scheme [2], the contractile force of ERM can cause ectopic inner foveal layer (EIFL) and distort retinal microvasculature configuration and can affect the photoreceptor layer, resulting in central vision loss and metamorphopsia. Pars plana vitrectomy with membrane peeling is the most widely accepted surgical approach to relieve symptoms and release the contraction [1]. However, because of the occult progression of ERM, visual acuity will not be substantially affected until significant pathologic changes have occurred. Structural factors found on optical coherence tomography (OCT) and optical coherence tomography angiography (OCTA), including central foveal thickness (CFT) [2], disruption of ellipsoid zone (EZ) integrity [3], tractional cystoid macular edema [4], “central foveal bouquet” [5], and the vessel density (VD) and perfusion density (PD) of foveal area can be increased [6] by ERM, and the presence of EIFL [2] has been shown to increase the risk of reduced visual acuity in eyes with ERM. Considering that the anatomical parameters of retinal layer thickness and retinal microvasculature might be affected by ERM, it has also been shown that EIFL in eyes with ERM had lower reproducibility of retinal layer thickness measurements [7] and higher retinal vessel and PD in the foveal area [6].

Microperimetry is a noninvasive tool for quantitatively detecting functional changes while evaluating photoreceptor function [8]. Microperimetry has been used to evaluate rod function in the early stage of age-related macular degeneration [9], assess disease severity in retinitis pigmentosa [8], evaluate the efficacy of surgery for ERM both pre- and post-operation [10], and so on. As a fundus-guided light sensitivity assessment, microperimetry combined with OCT and OCTA will be helpful for monitoring changes in visual function and retinal morphology over time, as well as for determining surgical indications. To our knowledge, there is little research that has analyzed the topographical correlation between microperimetric retinal sensitivity and structural factors in eyes with ERM.

The purpose of this study is to elucidate the topographic associations and relationships between macular structure and function in participants with idiopathic ERM related to EIFL and identify potential risk factors for disease progression.

## Methods

### Patients

A cross-sectional, observational chart review of patients diagnosed with unilateral idiopathic ERM at the First Affiliated Hospital of Sun Yat-Sen University between June 2022 and January 2024 was performed. All procedures in this project adhered to the tenets of the Declaration of Helsinki and were approved by the Ethics Committee of the First Affiliated Hospital of Sun Yat-Sen University (2024-429). Subjects had to read and give informed consent after having been informed of the objectives and methods of the research project. The inclusion criterion was the presence of a hyperreflective membrane at the vitreoretinal interface over the fovea detected with spectral-domain optical coherence tomography (SD-OCT) images, meeting the definition of idiopathic ERM [2]. All ERM patients were classified into two groups according to the staging scheme by Govetto et al. [2]: individuals without EIFL defined as Group 1 and with EIFL defined as Group 2. Exclusion criteria included the presence of any other ocular, systemic, or neurologic conditions that might affect retinal assessment, poor-quality multimodal image attributable to eye movement or media opacity, and previous intraocular surgery, excluding uncomplicated phacoemulsification. Best-corrected visual acuity (BCVA) was recorded and converted into logarithm of the minimal angle of resolution (logMAR) for further statistical analysis. The symptoms of metamorphopsia were measured by the Amsler Grid Test. Patients were required to be 40 to 85 years of age and have a refractive error of no more than − 4.00 D.

### Multimodal imaging

**Fig. 1:**
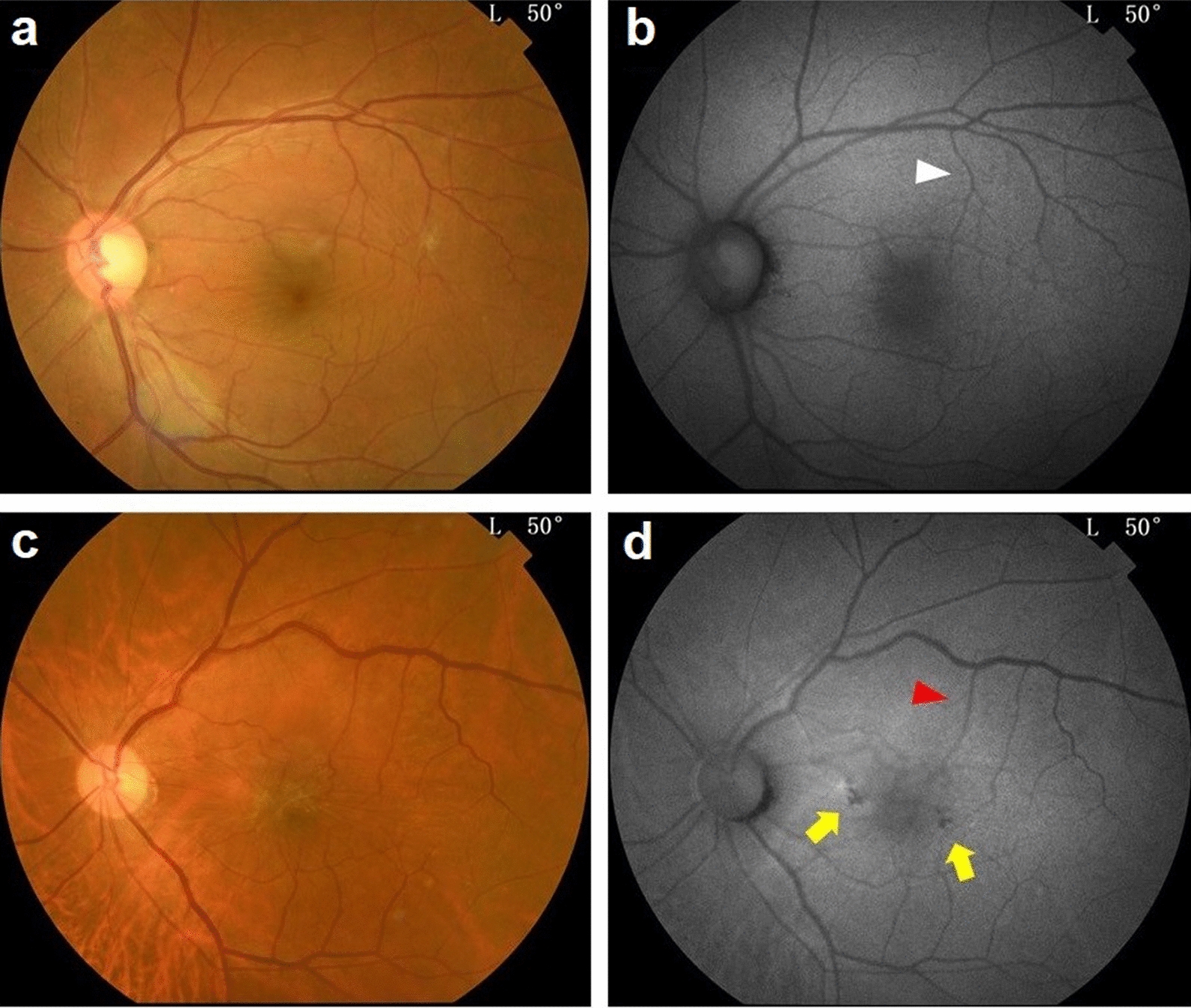
Representative retinal imaging of idiopathic epiretinal membrane (ERM). **a, b** Representative color fundus photography (CFP) and fundus autofluorescence (FAF) images of idiopathic ERM in Group 1 without ectopic inner foveal layer (EIFL). White arrowhead represents a retinal vessel branch strained by ERM. **c, d** Representative CFP and FAF images of idiopathic ERM in Group 2 with EIFL. Red arrowhead represents the distorted retinal vessel branch strained by tractional forces of ERM in EIFL eyes. Yellow arrows show the feeble spots of autofluorescence near macular fovea

Idiopathic ERMs were assessed by multimodal imaging using fundus-guided scotopic microperimetry, color fundus photography (CFP), fundus autofluorescence (FAF), SD-OCT, and OCTA. The schedule of assessments and testing were as follows: medical history was obtained, including age of onset and the extent of metamorphopsia, BCVA, CFP, FAF, Microperimetry, SD-OCT and OCTA were obtained following pupil dilation with one drop of 1% tropicamide. Macular-centered CFP and FAF were obtained by TOPCON (TRC-50DX, IA) fundus camera with a minimum resolution of 1200 × 1200 pixels. Figure 1a–d shows a CFP and FAF example from Group 1 (non-EIFL) and Group 2 (EIFL). All tests were performed by the same examiners strictly respecting standardized procedures.

### Microperimetry imaging and grading

Fundus-guided scotopic microperimetry using Nidek-MP3 (NAVIS-EX 1.8.0, NIDEK Technologies) was performed under dim light conditions following pupillary dilation. All patients underwent dark adaptation for at least 20 min prior to the test and completed a two-minute, uniform training session before assessment. A standardized testing protocol performed by Hariri et al. was used [11]. The exclusion criterion for fixation stability, assessed using the bivariate contour ellipse area, was defined as a fixation loss rate exceeding 30% [8]. The full test involved a 10° circular grid centered on the macula, containing 40 spots. The distribution of mean sensitivity (MS) at different ranges was characterized as follows: the foveal area sensitivity (MS_foveal_) was determined by the MS of 2° stimuli encompassing 8 spots. The parafoveal sensitivity (MS_parafoveal_) was considered as the MS of 6° and 10° stimuli covering 32 spots. The overall macular sensitivity (MS_macular_) was derived from the MS of 40 spots, assessed using 2°, 6°, and 10° stimuli. A mean macular sensitivity of less than 25 dB was considered abnormal [8, 12].

### OCT imaging and grading

SD-OCT horizontal and vertical B-scan consisting of 100-frames using automatic real-time tracking set and centered on the fovea were obtained using a Heidelberg Spectralis OCT unit (Heidelberg Engineering GmbH, Heidelberg, Germany). The presence or absence of EIFL was determined as presence of inner retina layer ectopic in the fovea [2, 13]. EZ disruption was considered as focal absence of the EZ [3]. CFT, in micrometers, was measured automatically in the Heidelberg Spectralis software. Two operators (YHS and FFY) independently identified EZ disruption on OCT images, and any disagreements in their evaluations were then reviewed by another experienced retinal specialist (PXW).

### OCTA imaging and AngioTool analysis

OCTA was obtained using the AngioPlex Cirrus 5000 HD-OCT system (Carl Zeiss AG, Oberkochen, Germany). The en face OCTA images centered on the fovea was performed with a 3 × 3 mm area scanning protocol [6] in each eye. The morphology and distribution of superficial and deep microvasculature were automatically generated. The foveal area was a central circle with a diameter of 1 mm, the parafoveal area was the sum area of four quadrant sectors and the macular area was the sum of a 3 × 3 mm circle of ETDRS area. The superficial VD and PD of each area were quantitatively measured using the AngioPlex software OMAG algorithm. Foveal avascular zone (FAZ) was measured manually utilizing 3 × 3 mm whole microcapillary OCTA images with the polygon drawing tool using ImageJ (ImageJ version 1; National Institutes of Health, Bethesda, MD) after setting the scale bar [14]. Two operators (QQW and CYW) measured FAZ area on OCTA images manually, and a third senior reader reviewed all FAZ area measurements and corrected the results when needed.

**Fig. 2:**
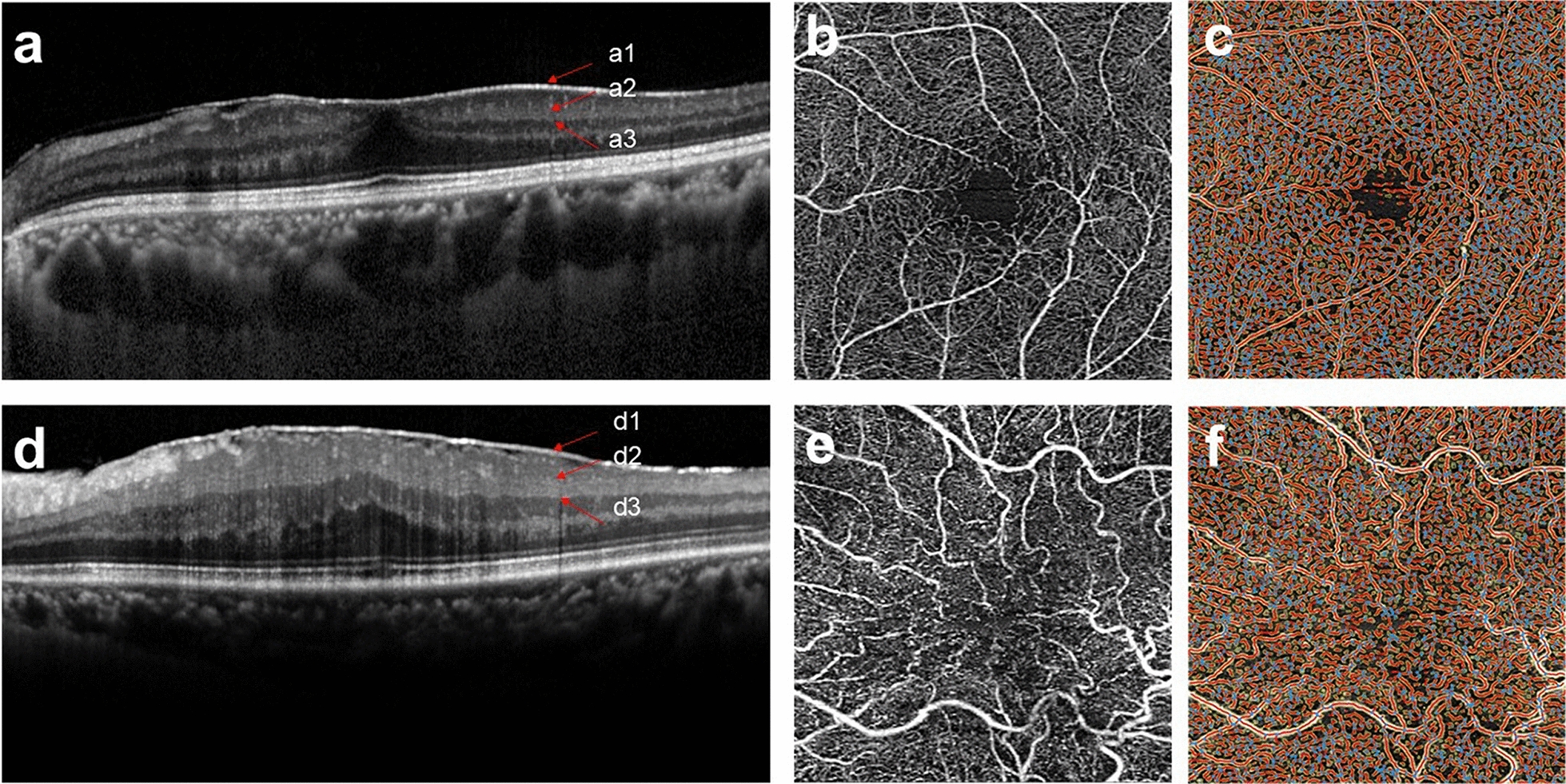
Retinal vascular layers displayed by segmenting retinal architecture in optical coherence tomography (OCT) structural images, projected on optical coherence tomography angiography (OCTA) images and processed by AngioTool. **a** En face OCT images of idiopathic epiretinal membrane (ERM) image without ectopic inner foveal layer (EIFL). a1 indicates the internal limiting membrane (ILM); a2 indicates the inner plexiform layer (IPL); a3 shows the IPL-INL boundary. **b, c **En face OCTA images of retinal vasculature and AngioTool software processed images of ERM without EIFL. **d **En face OCT images of idiopathic ERM image with EIFL. d1 indicates the ILM; d2 indicates the IPL; d3 indicates the IPL-INL boundary. **e **En face OCTA images of distorted retinal vasculature of ERM with EIFL. **f **En face OCTA images of distorted retinal vasculature processed using the AngioTool software

The 3 × 3 mm OCTA images of the whole layer, including superficial and deep vessels, were further processed after setting the scale bar by AngioTool 0.6 software [15] to obtain multiple indices. Figure 2a–f shows retinal segment layers in OCT images, projected on OCTA images and processed by AngioTool for Group 1 (non-EIFL) and Group 2 (EIFL).

### Microperimetry-OCTA overlays

**Fig. 3:**
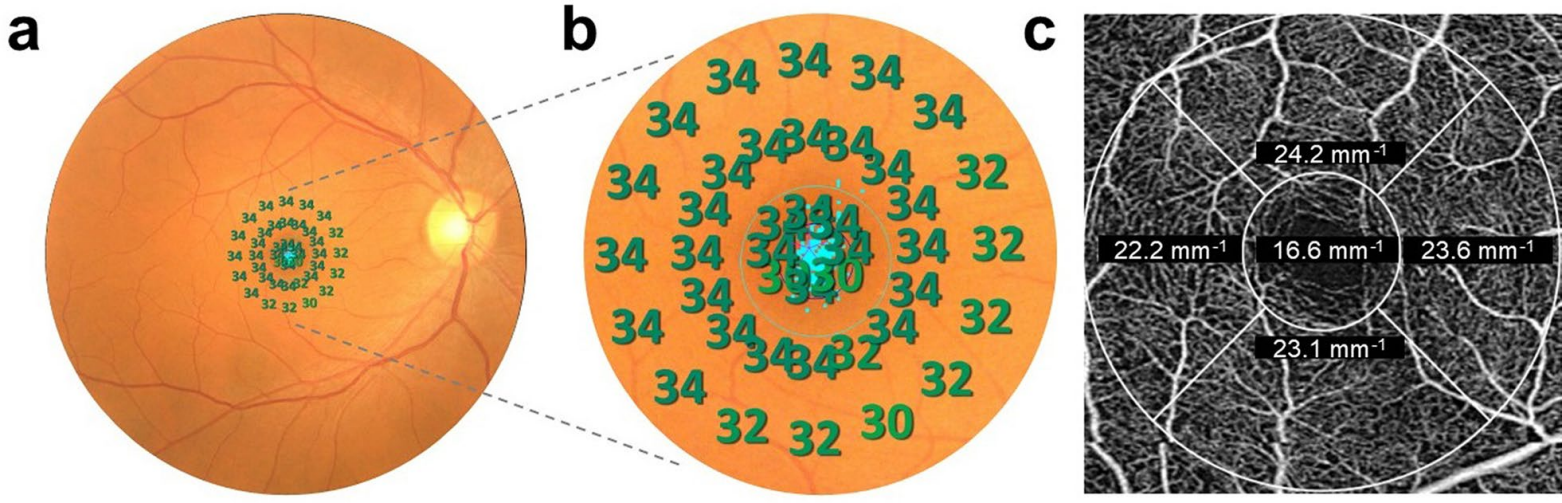
The distribution and correlation between macular microperimetry and optical coherence tomography angiography (OCTA). **a** Intact 10° microperimetry area mapped onto the color fundus photography (CFP) simultaneously acquired during microperimetry imaging. **b, c** Central enlarged microperimetry with the corresponding microvascular profile as obtained from OCTA 3 × 3 mm map demonstrating structure-function correlation. The central circle area covered by 2° stimuli in microperimetry corresponds to a 1 × 1 mm central area in the OCTA. The points in the middle circle covered by the 6° stimuli correspond to the total area of the four quadrant sectors. The overall macular sensitivity, calculated as the mean threshold of 40 points within the central 10° field, corresponds to the mean vascular density of the 3 × 3 mm area covered by the OCTA scan

To overlay a fundus-guided microperimetry sensitivity grid and the OCTA retinal microvascular image, a topographical correlation was developed between infrared fundus images acquired in microperimetry with OCTA images. The Microperimetry-OCTA overlays were correlated from angles and distance from the fovea as follows: the MS_foveal_ corresponded with OCTA_foveal_; MS_parafoveal_ corresponded to OCTA_parafoveal_; and MS_macular_ corresponded to OCTA_macular_ [9]. Figure 3a–c shows an example of a Microperimetry-OCTA overlay. The MS_foveal_ area covered by 2° stimuli in microperimetry corresponds to a 1 × 1 mm OCTA_foveal_ in OCTA. The MS_parafoveal_ points in the middle circle covered by the 6° stimuli correspond to the total area of the four quadrant sectors in OCTA_parafoveal_. The overall macular sensitivity, calculated as the mean threshold of 40 points within the central 10° field named MS_macular_, corresponds to the mean vascular density of the 3 × 3 mm OCTA_macular_ area covered by the OCTA scan.

### Statistical analyses

Statistical analyses were conducted utilizing R (version 4.3.1, R Foundation for Statistical Computing). Continuous data with a normal distribution was presented as mean and standard deviation, while non-normally distributed data was shown as median and interquartile ranges. Descriptive analysis and frequency calculations were done for categorial variables using the Chi-squared test. To investigate the relationship between foveal sensitivity and the variables under consideration, LASSO regression and quantile regression analyses were conducted. LASSO regression was employed utilizing linear regression with foveal sensitivity as the dependent variable. To further assess the relationship between foveal sensitivity and significant risk factors, quantile regression analysis was performed. This method models the impact of covariates on the conditional quantile of a response variable and was found to be more resilient compared to a generalized linear model. A *P* value of less than 0.05 indicates statistical significance.

## Results

### Study population

A total of 40 eyes in 40 patients (11 male and 29 female) with idiopathic ERM were enrolled in this study: 22 in Group 1 without EIFL and 18 in Group 2 with EIFL. The mean age was 63.55 ± 9.97 years in Group 1 and 65.78 ± 8.2 years in Group 2, respectively (*P* = 0.586). The age, sex, laterality and metamorphopsia were not significantly different between two groups. The BCVA was 0.06 ± 0.11 logMAR and 0.47 ± 0.30 logMAR in Group 1 and Group 2, respectively (*P* < 0.001; Table 1).

**Table 1:** Demographics and patient characteristics in each group

Variables	Total (n = 40)	Group 1 (n = 22)	Group 2 (n = 18)	*P* value
Age	64.55 ± 9.17	63.55 ± 9.97	65.78 ± 8.20	0.586
Sex, n (%)				0.498
Male	11 (28)	5 (23)	6 (33)	
Female	29 (72)	17 (77)	12 (67)	
Laterality, n (%)				1
Right	21 (52)	12 (55)	9 (50)	
Left	19 (48)	10 (45)	9 (50)	
BCVA	0.25 ± 0.30	0.06 ± 0.11	0.47 ± 0.30	**< 0.001**
Metamorphopsia, n (%)				0.240
No	22 (55)	14 (63.64)	8 (44.44)	
Yes	18 (45)	8 (36.36)	10 (55.56)	

*BCVA* = best-corrected visual acuity

The data are shown as mean ± standard deviation unless otherwise indicated

Age, sex, laterality, metamorphopsia differences between Groups 1 and 2 with ERM were assessed using the Chi-squared test

*P* values that are statistically significant are in bold font

### Macular sensitivity impacted by EIFL

**Fig. 4:**
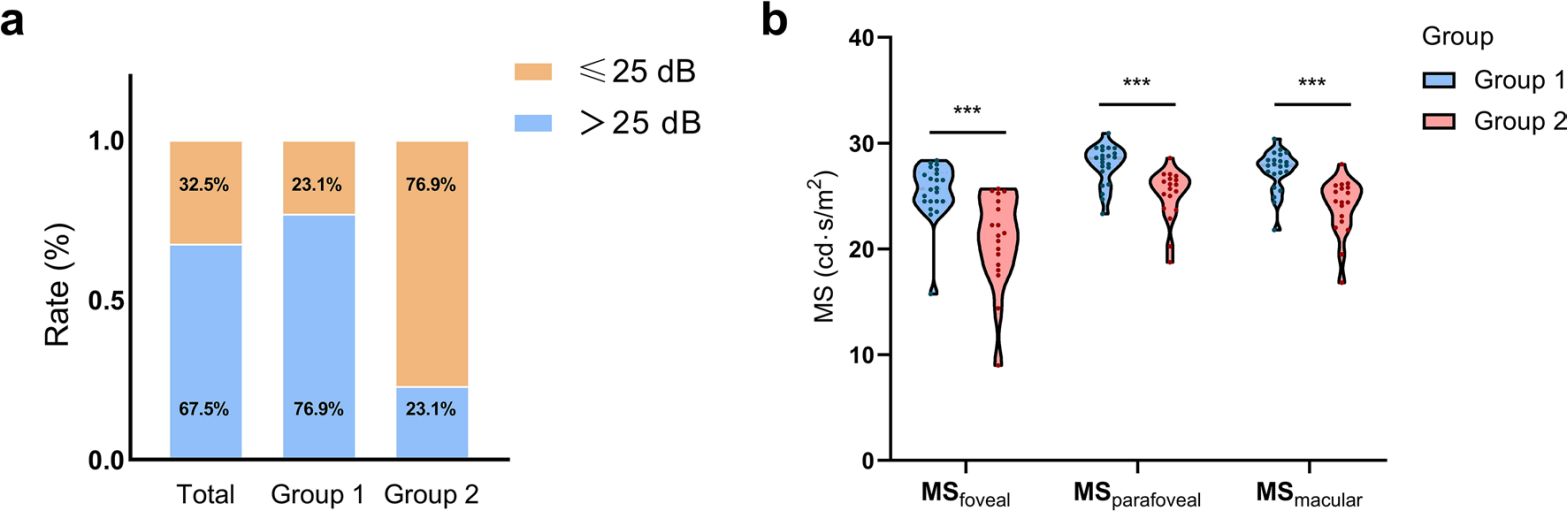
Low macular sensitivity distribution and sensitivity parameters comparison in microperimetry. **a** Percentile chart of low sensitivity percentage in total, Group 1, and Group 2. The percentage of low sensitivity of mean retinal sensitivity ≤ 25 dB (orange area) are plotted on the bar. **b** The comparison of retinal sensitivity for each area is found along the x-axis. The difference between the two groups is plotted (***, *P* < 0.001). MS, mean sensitivity

All subjects were able to perform the scotopic microperimetry successfully, yielding a mean scotopic MS_macular_ of 26.05 (24.40–28.00) dB in total and 27.90 (27.13–28.40) dB and 24.45 (22.73–25.78) dB in Groups 1 and 2, respectively (*P* < 0.001). According to the cutoff point for the definition of low macular sensitivity [8] in this study, MS_macular_ below 25 dB was found in 23.1% and 76.9% of Groups 1 and 2, respectively (*P* = 0.0073) (Fig. 4a). To detect more subtle variations in light sensitivity, we compared the foveal and parafoveal area sensitivity affected by EIFL separately. The MS_foveal_ was 25.69 (24.50–27.09) dB and 21.38 (18.75–24.31) dB in Groups 1 and 2, respectively (*P* < 0.001). The MS_parafoveal_ was 28.56 (27.43–29.27) dB and 25.88 (24.11–26.59) dB in Groups 1 and 2, respectively (*P* < 0.001; Fig. 4b).

### Morphological alteration of ectopic inner retinal layer and distorted macular vessels

To assess the morphological alteration caused by idiopathic ERM traction, we quantified the retinal morphology from OCT and structural parameters of the macular microvasculature from OCTA. Table 2 presents a comparison of the results obtained by OCT and OCTA between the two groups. The presence of EZ disruption (9% vs. 56%) was significantly different between the two groups (*P* = 0.004). Mean CFT was 262.00 (227.75–333.75) µm and 570.00 (479.00–593.25) µm, and FAZ area was 0.17 (0.13–0.25) mm^2^ and 0.04 (0.01–0.07) mm^2^ in Groups 1 and group 2, respectively, which showed significant differences (all *P* < 0.001).

**Table 2:** Multimodal imaging assessing macular structure and microvessel parameters in each group

Variables	Total (*n* = 40)	Group 1 (n = 22)	Group 2 (n = 18)	*P* value
OCT parameters				
EZ, n (%)				**0.004**
Integrity	28 (70)	20 (91)	8 (44)	
Disruptive	12 (30)	2 (9)	10 (56)	
CFT (µm)	380.50 (252.25, 517.75)	262.00 (227.75, 333.75)	570.00 (479.00, 593.25)	**< 0.001**
OCTA parameters				
FAZ (mm^2^)	0.08 (0.05, 0.23)	0.17 (0.13, 0.25)	0.04 (0.01, 0.07)	**< 0.001**
VD_macular_ (mm^−1^)	21.00 (20.35, 22.10)	21.05 (20.47, 22.03)	20.95 (20.27, 21.98)	0.634
VD_parafoveal_ (mm^−1^)	21.70 (20.75, 22.42)	22.10 (21.38, 22.78)	21.30 (20.60, 22.00)	**0.041**
VD_foveal_ (mm^−1^)	16.60 (13.57, 20.15)	13.90 (11.22, 16.60)	20.20 (16.75, 21.58)	**< 0.001**
PD_macular_ (%)	0.39 (0.37, 0.40)	0.39 (0.37, 0.39)	0.39 (0.38, 0.41)	0.169
PD_parafoveal_ (%)	0.40 (0.39, 0.41)	0.40 (0.38, 0.41)	0.40 (0.39, 0.41)	0.703
PD_foveal_ (%)	0.30 (0.23, 0.36)	0.25 (0.20, 0.29)	0.36 (0.31, 0.40)	**< 0.001**
AngioTool analysis				
Vessel area (mm^2^)	4.08 (3.96, 4.17)	4.11 (3.97, 4.20)	4.06 (3.93, 4.14)	0.283
Vessels percentage area (%)	45.47 (44.06, 46.38)	45.71 (44.17, 46.71)	45.22 (43.76, 46.07)	0.283
Total vessels length (mm^−1^)	192.71(185.28, 201.64)	200.01 (192.48, 204.83)	187.13 (173.89, 192.71)	**0.002**
Average vessels length (mm^−1^)	1.02 (0.76, 1.33)	1.23 (0.95, 1.45)	0.86 (0.57, 1.02)	**0.007**
Total number of junctions (n)	1923.00 (1767.50, 2157.50)	2138.00 (1896.00, 2207.75)	1784.00 (1523.75, 1923.00)	**< 0.001**
Junction density (mm^−1^)	214.10 (196.79, 240.21)	238.04 (211.11, 245.85)	198.63 (169.64, 214.10)	**< 0.001**
Total number of end points (n)	1330.00 (1177.75, 1466.75)	1229.50 (1143.00, 1351.75)	1426.50 (1330.00, 1552.00)	**0.002**

*EZ* = ellipsoid zone; *CFT* = central foveal thickness; *FAZ* = foveal avascular zone; *VD* = vessel density; *PD* = perfusion density

Data are presented as median (1st quartile, 3rd quartile) for skewed distributed variables, and number (%) for categorical variables. When comparing differences between two groups, the Chi-squared test was used for categorical variables and the Mann-Whitney U test was used for skewed variables

Statistically significant differences are in bold font

In Group 1 and Group 2, VD_foveal_ was 13.90 (11.22–16.60) mm^−1^ and 20.20 (16.75–21.58) mm^−1^ respectively; PD_foveal_ was 0.25 (0.20–0.29)% and 0.36 (0.31–0.40) %, respectively. Both showed significant differences (all *P* < 0.001). The VD_parafoveal_ was 22.10 (21.38–22.78) mm^−1^ and 21.30 (20.60–22.00) mm^−1^ between Groups 1 and 2, showing statistical significance (*P* = 0.041). Additionally, the differences in PD_parafoveal_, VD_macular_ and PD_macular_ were not significant at the cutoff of *P* ≤ 0.05 (Table 2).

### Factors associated with macular microcapillary characteristics

Table 2 presents the comparisons of results obtained from OCTA images assessed using the AngioTool software between Groups 1 and 2. In Group 2, the EIFL population had shorter ‘total vessels length’ of 187.13 (173.89–192.71) mm^−1^ compared with 200.01 (192.48–204.83) mm^−1^ in Group 1 (*P* = 0.002). Shorter ‘average vessels length’ of 0.86 (0.57–1.02) mm^−1^ in Group 2 was observed compared to 1.23 (0.95–1.45) mm^−1^ in Group 1 (*P* = 0.007). Group 2 showed a lower ‘total number of junctions’ of 1784.00 (1523.75–1923.00) compared to Group 1 with 2138.00 (1896.00–2207.75) (*P* < 0.001). Group 2 also had a lower ‘junctions density’ of 198.63 (169.64–214.10) mm^−1^ compared with 238.04 (211.11, 245.85) mm^−1^ in Group 1 (*P* < 0.001). Conversely, the ‘total number of end points’ was higher in Group 2 with 1426.50 (1330.00, 1552.00) compared to 1229.50 (1143.00, 1351.75) in Group 1 (*P* = 0.002). The difference in ‘vessels area’ or ‘vessels percentage area between the groups was not statistically significant.

### LASSO regression analysis to identify structure and function parameters associated with foveal sensitivity

**Fig. 5:**
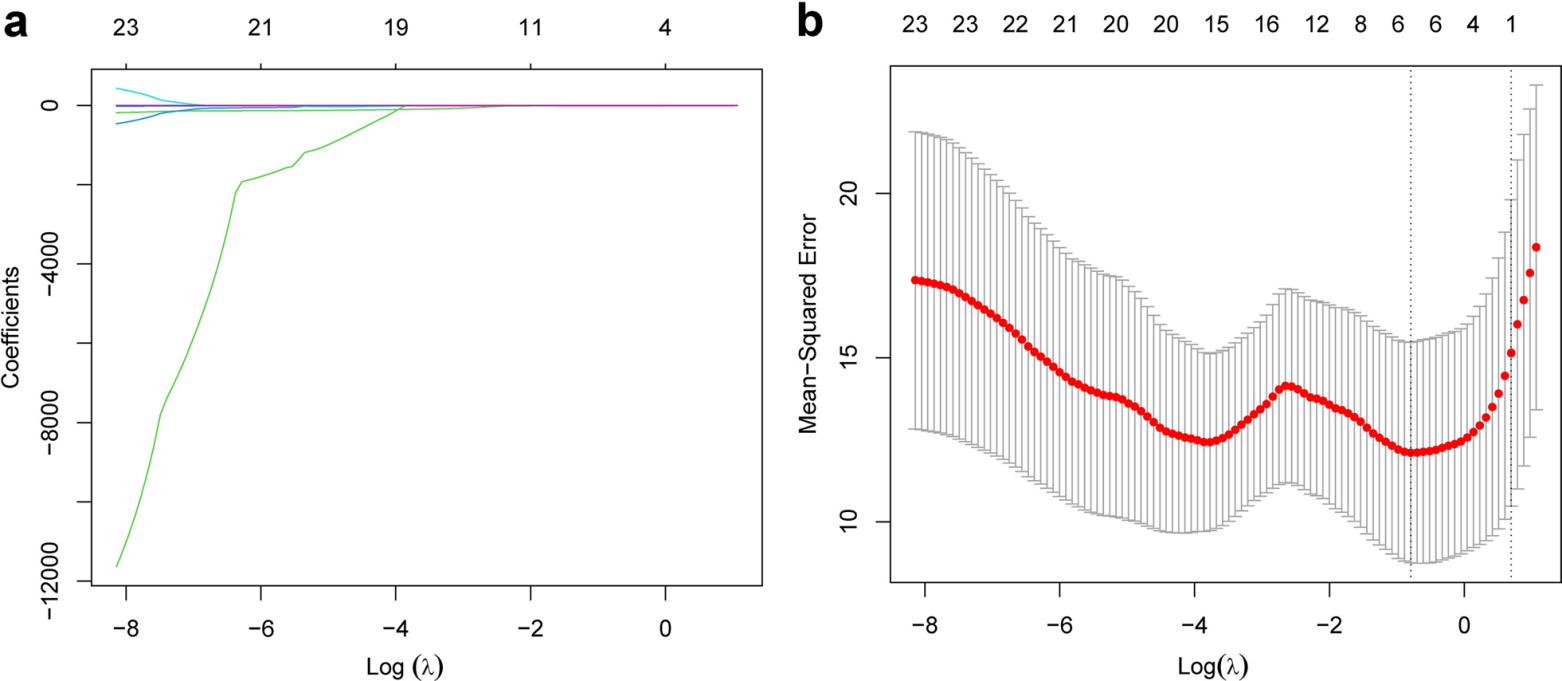
LASSO regression analysis showed Log(λ) = − 0.18303 when the error of the model is minimized, and five variables were selected for further analysis. **a, b** The LASSO coefficient spectrum was analyzed for five variables associated with foveal light sensitivity. Each curve in the spectrum represents a specific variable

Based on univariate LASSO regression analysis, five variables including EIFL, BCVA, EZ, CFT, VD_parafoveal_, were significantly associated with MS_foveal_ in patients with ERM (Fig. 5). We utilized ten-fold cross-validation to select the penalty term, lambda (λ). Log(λ) = − 0.18303 (λ = 0.6561) when the error of the model is minimized, and five variables (i.e., EIFL, BCVA, EZ, CFT, VD_parafoveal_) were selected for further quantile regression analysis.

### Quantile regression analysis identifying VDparafoveal as positively correlating with foveal sensitivity

**Fig. 6:**
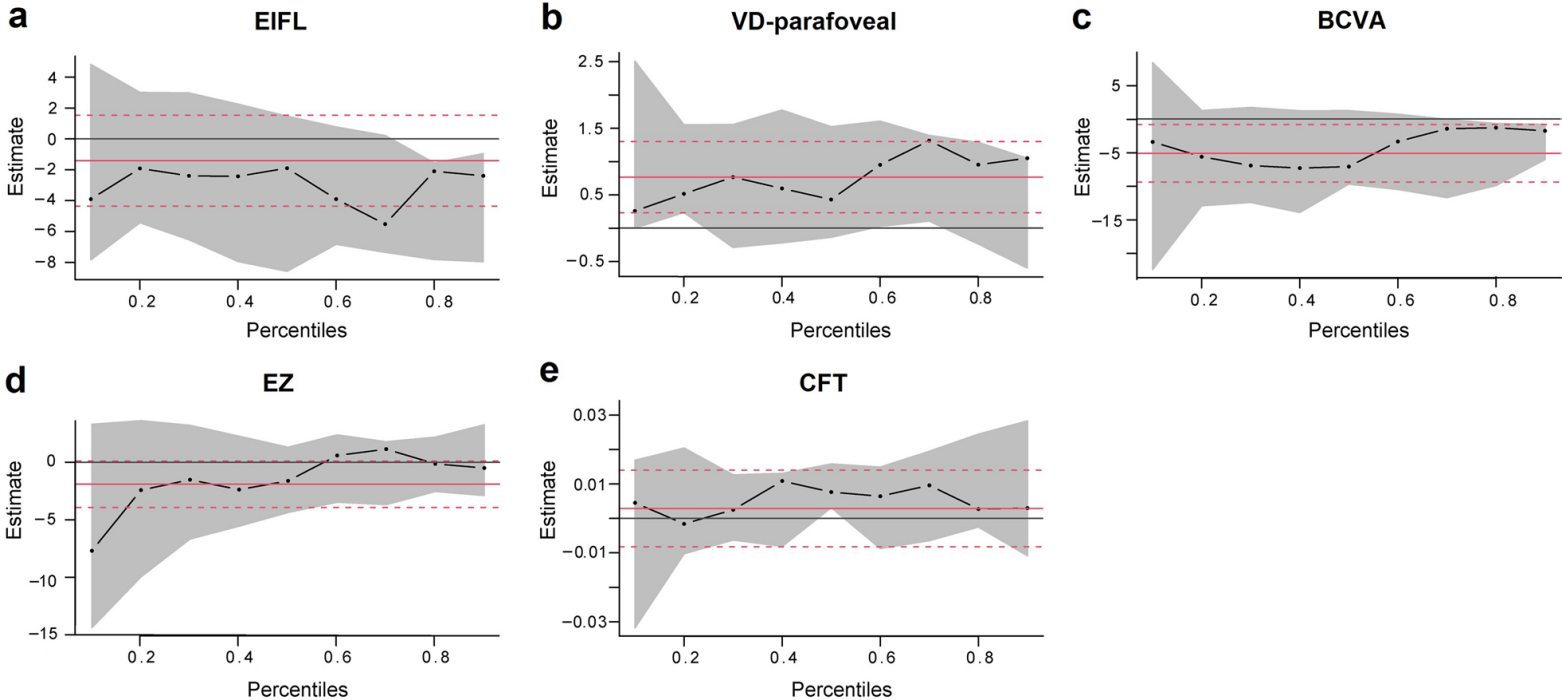
Estimated parameters by quantiles with 95% confidence intervals. **a–e** Quantile regression plots for covariates in the final model, including EIFL, VD_parafoveal_, BCVA, EZ and CFT. The x- and y-axis denote the quantile scale and the effect of a covariate on foveal sensitivity for a given quantile, respectively. The broken black line represented the estimated coefficients and the gray area represented 95% confidence interval of the corresponding parameters. EIFL, ectopic inner foveal layer; VD-parafoveal, vessel density–parafoveal; BCVA, best-corrected visual acuity; EZ, ellipsoid zone; CFT, central foveal thickness

Table 3 presents the plots generated from quantile regression analysis for five covariates i.e., EIFL, BCVA, EZ, CFT and VD_parafoveal_. Foveal light sensitivity was positively correlated with VD_parafoveal_ and negatively correlated with EIFL alteration. Furthermore, VD_parafoveal_ had a larger effect on MS_foveal_ than any other quantitative covariates as seen from the overall pattern in Fig. 6.

**Table 3:** Quantile regression analysis indicates the overall pattern of five covariates

Variates	*P*_10_	*P*_20_	*P*_30_	*P*_40_	*P*_50_	*P*_60_	*P*_70_	*P*_80_	*P*_90_
EIFL	−3.899	−1.916	−2.394	−2.427	−1.898	−3.900^*^	−5.522^*^	−2.099	−2.391
BCVA	−3.418	−5.630	−6.943	−7.331	−7.098	−3.365	−1.461	−1.300	−1.762
EZ disruptive	−7.698	−2.423	−1.524	−2.382	−1.621	0.585	1.138	−0.137	−0.506
CFT	0.005	−0.001	0.002	0.011	0.008	0.006	0.010	0.003	0.003
VD_parafoveal_	0.256	0.513	0.764	0.594	0.428	0.949*	1.311***	0.950***	1.048

*EIFL* = ectopic inner foveal layer; *BCVA* = best-corrected visual acuity; *EZ* = ellipsoid zone; *CFT* = central foveal thickness; *VD* = vessel density

Statistically significant differences are in bold font. *P<0.05, **P<0.01, ***P<0.001

## Discussion

Here, we present the baseline data of retina macular microperimetry, OCT and OCTA in idiopathic ERM, and the relationships between macular focal sensitivity with retinal microstructure and microvascular parameters in ERM patients related to EIFL. ERM patients with EIFL exhibited significantly reduced macular sensitivity in MS_macular_ compared to those without EIFL, as well as in MS_foveal_ and MS_parafoveal_. Additionally, ERM patients with EIFL demonstrated significantly increased VD_foveal_ and PD_foveal_ compared to patients without EIFL; however, the VD_parafoveal_ was significantly decreased. LASSO and Quantile regression analysis revealed that increased VD_parafoveal_ positively influenced the light sensitivity of MS_fovea_, whereas the presence of EIFL negatively impacted the light sensitivity of MS_fovea_ in patients with ERM.

Considering the possibility of participants’ fatigue, minimal learning effect, and similar repeatability [8, 16] of microperimetry results during the test, we adopted a short training and practice model to conduct microperimetry. Thus, our results suggest that microperimetry not only showed results consistent with the trends of visual acuity but also demonstrated subregional effects of the ERM on the local macular area. Feng and colleagues [10] recently analyzed MP-1 microperimetry variation in a cohort of 30 patients with ERM who underwent pars plana vitrectomy surgery. Using a grid with a diameter of 10°, they noted a rising trend in macular sensitivity after ERM peeling surgery, suggesting that ERM in the macula can decrease macular sensitivity.

Our results indicated that macular sensitivity was significantly lower in ERM patients with EIFL than in those without EIFL preoperatively. The topographic assessment provided by microperimetry testing in this study revealed that stabilized and undisturbed anatomical structure of the inner retinal layer play an important role in maintaining the stability of macular sensitivity, not only in the foveal area, but also in the parafoveal area.

The unique anatomical configuration of the fovea and parafovea results from the displacement of centrally distributed photoreceptors combined with the ‘Müller cell cone’ and the ‘z-shaped’ pattern of parafoveal Müller cells [17, 18]. The presence of ERM may exert both centrifugal and contractile force on the macular surface, leading to deformation of the inner retinal layer. Additionally, the occurrence of EIFL may disrupt the balance maintained by Müller cells, resulting in the disorganization of the photoreceptor layer. Previous studies have identified that factors such as disruption of EZ integrity [3], central macular thickness [2], and alterations in photoreceptor outer segment length [19] correlated with preoperative and postoperative visual acuity. In this study, we identified disruption of EZ integrity and increased CFT as risk factors that correlate with microperimetry, which is consistent with the findings related to visual acuity. The observed increased CFT and disruption of EZ integrity were statistically associated with a decline in macular sensitivity. The decline may not only be attributed to the disruption of the balance of vertical and horizontal forces by Müller cells, but also to the exacerbation of Müller cells activation.

The ERM not only affects inner retinal layer microanatomy, but also impacts the inner retinal layer microvasculature. Here, the OCTA analysis in eyes with ERM showed increased VD and PD in the foveal area along with a statistical decline in microperimetry parameters, demonstrated that retinal microvasculature density in the foveal area is a determining factor for light sensitivity. Physiologically, the diaphaneity of fovea tissue in the foveal pit, which overlies the photoreceptors, assures specialization for high visual acuity [18]. The presence of ERM significantly altered the FAZ, varying from a capillary-free zone to an area of near-complete obliteration of the FAZ in ERM eyes with EIFL. The increased microvasculature in the foveal area may cause light scattering and absorption by blood vessels, thereby blocking incoming light and resulting in a reduction in visual sensitivity [20]. This hypothesis was also validated by studies in age-related macular degeneration and Macular Telangiectasia [21, 22].

In this study, decreases in ‘total vessel length’, ‘average vessel length’, ‘total number of junctions’, and ‘junctions density’ were found in ERM eyes with EIFL. These parameters represent the division of the lengths and branch junctions of all vessels. Thus, the decrease in vessel length and branch junctions indicates that the vessels become more linear in shape, and the traction force by ERM made the vessels less tortuous. In tandem, Miyazawa et al. [23] quantified the macular vessel tortuosity with ERM following surgery and indicated that it was associated with visual outcomes after surgery. Moreover, Feng et al. [10] demonstrated that the release of ERM traction with surgery could improve postoperative visual results, indicating that increasing the parafoveal VD through the release of these taut vessels would be beneficial for light sensitivity.

Our study illustrated that VD_parafoveal_ depicts a positive effect on MS_foveal_, suggesting that a higher concentration of microvasculature in the parafoveal area correlates with increased foveal sensitivity. From an anatomical point of view, the blood vessel parameter analysis using Angiotool revealed that vessel length and junction density decreased under the influence of ERM with EIFL. From the perspective of retinal metabolism, under physiological conditions, Müller cells in the central fovea tissue not only provide structural support to the fovea but also facilitate functional and metabolic interactions with photoreceptors [18]. However, under pathologic mechanical stress, such as that induced by the presence of ERM, retinal injury can activate Müller cells through various mechanisms and trigger reactive gliosis [24]. Therefore, we hypothesize that the observed positive correlation between VD_parafoveal_ and MS_foveal_ may not only be a phenomenon induced by ERM, but also could be a result of increased metabolic demands following morphological alterations in the fovea. The underlying pathophysiological mechanism contributing to VD_parafoveal_ and its positive effect on MS_foveal_ remain speculative; however, they may provide new insights into potential therapeutic strategies for ERM. This factor could influence surgical decision-making in ERM patients with EIFL.

The current study has several limitations. First, it was a baseline structure and function analysis in which no adjustments were made for multiple comparisons and no long-term observation took place. Factors like lifestyle (smoking, alcohol consumption), nutritional status, and environmental exposure could have been considered. Second, the accuracy of microperimetry depends on the participants’ mental state and cooperation, which could vary between patients or even within a patient over time. To avoid this bias, all patients completed a uniform two-minute training session prior to beginning the test, and we excluded the values obtained during the training. Third, metamorphopsia is one of the major symptoms in ERM patients, and it is better to quantitatively measure it using M-CHARTS [25] instead of the Amsler Grid Test, as was done in the present study.

## Conclusions

In conclusion, the present study allows the analysis of this issue from a different perspective i.e., increased VD_parafoveal_ of ERM patients correlates positively with foveal light sensitivity. Thus, increasing vessel perfusion may upgrade the visual light sensitivity projected on the cone photoreceptors. Further, the displacement of inner retinal layer may cause photoreceptor layer damage and deformation. Surgeons should consider using this structural biomarker when counseling patients and making a decision about the timing of surgery for ERM removal. Additional studies with a larger number of patients, a prospective design, and long-term follow-up are needed to explore a better understanding of pathologic mechanisms leading to the ERM with EIFLs.

## Data Availability

Data are available on reasonable request.

## Abbreviations

ERM: Idiopathic epiretinal membrane
EIFL: Ectopic inner foveal layer
SD-OCT: Spectral-domain optical coherence tomography
OCTA: Optical coherence tomography angiography
VD: Vessel density
PD: Perfusion density
CFT: Central foveal thickness
EZ: Ellipsoid zone
BCVA: Best-corrected visual acuity
logMAR: Logarithm of the minimal angle of resolution
CFP: Color fundus photography
FAF: Fundus autofluorescence
MS: Mean sensitivity
FAZ: Foveal avascular zone
λ: Lambda

## References

[1] Flaxel CJ, Adelman RA, Bailey ST, Fawzi A, Lim JI, Vemulakonda GA, et al. Idiopathic epiretinal membrane and vitreomacular traction preferred practice pattern^®^. Ophthalmology. 2020;127(2):145–83. 10.1016/j.ophtha.2019.09.022 31757497

[2] Govetto A, Lalane RA 3rd, Sarraf D, Figueroa MS, Hubschman JP. Insights into epiretinal membranes: presence of ectopic inner foveal layers and a new optical coherence tomography staging scheme. Am J Ophthalmol. 2017;175:99–113. 27993592 10.1016/j.ajo.2016.12.006

[3] Oster SF, Mojana F, Brar M, Yuson RM, Cheng L, Freeman WR. Disruption of the photoreceptor inner segment/outer segment layer on spectral domain-optical coherence tomography is a predictor of poor visual acuity in patients with epiretinal membranes. Retina. 2010;30(5):713–8. 20038861 10.1097/IAE.0b013e3181c596e3

[4] Fang IM, Hsu CC, Chen LL. Correlation between visual acuity changes and optical coherence tomography morphological findings in idiopathic epiretinal membranes. Graefes Arch Clin Exp Ophthalmol. 2016;254(3):437–44. 26016811 10.1007/s00417-015-3069-0

[5] Govetto A, Bhavsar KV, Virgili G, Gerber MJ, Freund KB, Curcio CA, et al. Tractional abnormalities of the central foveal bouquet in epiretinal membranes: clinical spectrum and pathophysiological perspectives. Am J Ophthalmol. 2017;184:167–80. 29106913 10.1016/j.ajo.2017.10.011

[6] Yu HY, Na YJ, Lee SC, Lee MW. Characteristics of the macular microvasculature in idiopathic epiretinal membrane patients with an ectopic inner foveal layer. Retina. 2023;43(4):574–80. 36728890 10.1097/IAE.0000000000003710

[7] Jung I, Na YJ, Lee SC, Lee MW. Reproducibility of each retinal layer thickness measurement in epiretinal membrane patients with ectopic inner foveal layers. Eye Vis (Lond). 2023;10:3. 36597171 10.1186/s40662-022-00321-2 PMC9811706

[8] Lad EM, Duncan JL, Liang W, Maguire MG, Ayala AR, Audo I, et al. Baseline microperimetry and OCT in the RUSH2A study: structure-function association and correlation with disease severity. Am J Ophthalmol. 2022;244:98–116. 36007554 10.1016/j.ajo.2022.08.013 PMC9712171

[9] Nassisi M, Tepelus T, Corradetti G, Sadda SR. Relationship between choriocapillaris flow and scotopic microperimetry in early and intermediate age-related macular degeneration. Am J Ophthalmol. 2021;222:302–9. 32360341 10.1016/j.ajo.2020.04.018

[10] Feng J, Yang X, Xu M, Wang Y, Shi X, Zhang Y, et al. Association of microvasculature and macular sensitivity in idiopathic macular epiretinal membrane: using OCT angiography and microperimetry. Front Med (Lausanne). 2021;8:655013. 34869402 10.3389/fmed.2021.655013 PMC8635104

[11] Hariri AH, Tepelus TC, Akil H, Nittala MG, Sadda SR. Retinal sensitivity at the junctional zone of eyes with geographic atrophy due to age-related macular degeneration. Am J Ophthalmol. 2016;168:122–8. 27189929 10.1016/j.ajo.2016.05.007

[12] Rodrigues Neto TDS, Silva Neto EDD, Higashi AH, Megnis BP, Haddad MAO, Monteiro MLR, et al. Normative data for macular perimetry using the MP-3 microperimeter in healthy individuals. Arq Bras Oftalmol. 2023. 10.5935/0004-2749.2021-0472. 39298722 10.5935/0004-2749.2021-0472 PMC11622843

[13] González-Saldivar G, Berger A, Wong D, Juncal V, Chow DR. Ectopic inner foveal layer classification scheme predicts visual outcomes after epiretinal membrane surgery. Retina. 2020;40(4):710–7. 30829991 10.1097/IAE.0000000000002486

[14] Schneider CA, Rasband WS, Eliceiri KW. NIH Image to ImageJ: 25 years of image analysis. Nat Methods. 2012;9(7):671–5. 22930834 10.1038/nmeth.2089 PMC5554542

[15] Zudaire E, Gambardella L, Vermeren S. A computational tool for quantitative analysis of vascular networks. PLoS One. 2011;6(11):e27385. 22110636 10.1371/journal.pone.0027385 PMC3217985

[16] Alibhai AY, Mehta N, Hickson-Curran S, Moreira-Neto C, Levine ES, Reichel E, et al. Test-retest variability of microperimetry in geographic atrophy. Int J Retina Vitreous. 2020;6:16. 32377380 10.1186/s40942-020-00217-0 PMC7193411

[17] Bringmann A, Wiedemann P. Müller glial cells in retinal disease. Ophthalmologica. 2012;227(1):1–19. 21921569 10.1159/000328979

[18] Bringmann A, Syrbe S, Görner K, Kacza J, Francke M, Wiedemann P, et al. The primate fovea: structure, function and development. Prog Retin Eye Res. 2018;66:49–84. 29609042 10.1016/j.preteyeres.2018.03.006

[19] Shiono A, Kogo J, Klose G, Takeda H, Ueno H, Tokuda N, et al. Photoreceptor outer segment length: a prognostic factor for idiopathic epiretinal membrane surgery. Ophthalmology. 2013;120(4):788–94. 23290984 10.1016/j.ophtha.2012.09.044

[20] Agte S, Junek S, Matthias S, Ulbricht E, Erdmann I, Wurm A, et al. Müller glial cell-provided cellular light guidance through the vital guinea-pig retina. Biophys J. 2011;101(11):2611–9. 22261048 10.1016/j.bpj.2011.09.062 PMC3297812

[21] Weale RA. Why does the human retina possess a fovea? Nature. 1966;212(5059):255–6. 4961475 10.1038/212255a0

[22] Takayama K, Ooto S, Tamura H, Yamashiro K, Otani A, Tsujikawa A, et al. Retinal structural alterations and macular sensitivity in idiopathic macular telangiectasia type 1. Retina. 2012;32(9):1973–80. 22487581 10.1097/IAE.0b013e318251a38b

[23] Miyazawa K, Sakimoto S, Kanai M, Shiraki A, Takahashi S, Shiraki N, et al. Vascular tortuosity analysis in eyes with epiretinal membrane imaged by optical coherence tomography angiography. BMC Ophthalmol. 2022;22(1):198. 35501767 10.1186/s12886-022-02420-z PMC9063110

[24] Govetto A, Hubschman JP, Sarraf D, Figueroa MS, Bottoni F, dell’Omo R, et al. The role of Müller cells in tractional macular disorders: an optical coherence tomography study and physical model of mechanical force transmission. Br J Ophthalmol. 2020;104(4):466–72. 31326893 10.1136/bjophthalmol-2019-314245

[25] Yanagida K, Wakabayashi Y, Usui Y, Umazume K, Yamamoto K, Kawakami S, et al. Ectopic inner foveal layer as a factor associated with metamorphopsia after vitrectomy for epiretinal membrane. Acta Ophthalmol. 2022;100(7):775–80. 35076169 10.1111/aos.15092

